# Medium-Chain Triglycerides: Scientific and Regulatory Perspectives from Germany and Japan with a US Context—A Concise Review

**DOI:** 10.3390/nu18071027

**Published:** 2026-03-24

**Authors:** Christina Heidt, Heiko Oertling, Marie Abramowicz, Yuki Otsubo, Soyoka Tokunaga, Shogo Tsujino

**Affiliations:** 1 Department of Food Technology, Trier University of Applied Sciences, Schneidershof, 54293 Trier, Germany; h.oertling@blv.hochschule-trier.de (H.O.); m.abramowicz@vw.hochschule-trier.de (M.A.); 2 Faculty of Medicine, University of Muenster, 48149 Muenster, Germany; 3 Strategic Invention R&D, Technical Division, The Nisshin OilliO Group, Ltd., Yokohama 235-8558, Kanagawa, Japan; y-ohtsubo@nisshin-oillio.com (Y.O.); s-tokunaga@nisshin-oillio.com (S.T.); s-tsujino@nisshin-oillio.com (S.T.)

**Keywords:** medium-chain triglycerides, medium-chain fatty acids, β-oxidation, ω-oxidation, FSMP, FOSHU

## Abstract

Medium-chain triglycerides (MCTs, C6–C12 fatty acids) exhibit rapid absorption, preferential portal transport, efficient mitochondrial β-oxidation, promoting acetyl-CoA formation and ketogenesis. Under high lipid flux or impaired β-oxidation, MCTs undergo ω-oxidation, producing dicarboxylic acids further metabolized peroxisomally, preventing fatty acid accumulation. Industrially, MCTs are synthesized via chemical or enzymatic esterification of caprylic (C8) and capric (C10) acids, yielding high-purity triglycerides used in food and medical nutrition. In Germany and across the European Union, they are primarily used in Foods for Special Medical Purposes (FSMPs) for conditions such as fat malabsorption, ketogenic dietary therapy for refractory epilepsy, and inherited disorders of long-chain fatty-acid oxidation. In Japan, MCTs are additionally incorporated into functional food systems, including Foods for Specified Health Uses (FOSHU) and Foods with Function Claims (FFC), targeting generally healthy adults and older populations. In the United States, MCTs are widely marketed as food ingredients, dietary supplements, clinical nutrition products, and medical foods, reflecting their status as generally recognized as safe (GRAS). This review integrates knowledge on MCT metabolism, industrial production, clinical applications, and regulatory frameworks in Germany, Japan, and the United States, highlighting how regulatory environments influence the translation of MCTs from clinical nutrition toward broader preventive health strategies.

## 1. Introduction

Medium-chain triglycerides (MCTs) are primarily composed of medium-chain fatty acids (C6–C12) [1]. After ingestion, they are rapidly hydrolyzed and absorbed independently of bile acids and pancreatic lipase, with most transported via the portal vein to the liver [1]. In hepatocytes, MCTs undergo rapid mitochondrial β-oxidation, bypassing the carnitine-dependent transport required for long-chain fatty acids, which accelerates acetyl-CoA formation and ketogenesis [1]. Under conditions of high lipid flux or impaired β-oxidation, MCTs can also undergo ω-oxidation in the endoplasmic reticulum, producing dicarboxylic acids that are subsequently metabolized via peroxisomal β-oxidation, providing a metabolic safeguard against fatty acid accumulation [2,3].

These unique metabolic features underpin differing applications across countries. In Germany, MCTs are clinically established as foods for special medical purposes (FSMPs), targeting fat malabsorption, exocrine pancreatic insufficiency, short bowel syndrome, ketogenic diet-responsive neurological disorders, and long-chain fatty acid oxidation defects [4,5]. Clinical evidence supports improved energy provision, enhanced ketogenesis, and bypass of metabolic blockades [6]. In contrast, in Japan, MCTs are used not only for patients but also for healthy individuals as functional foods to maintain and promote their health by supporting energy metabolism and muscle function [7]. In the United States, MCTs are widely marketed as food ingredients and dietary supplements and are generally recognized as safe (GRAS), reflecting a regulatory environment that facilitates broad consumer use.

Regulatory frameworks reinforce these distinctions: Germany emphasizes therapeutic use under medical supervision, Japan promotes health maintenance through systems such as Foods for Specified Health Uses (FOSHU) and Foods with Function Claims, while the United States largely regulates MCTs within the food ingredient and dietary supplement markets.

Therefore, this review critically evaluates and contrasts the roles of MCTs in Germany, Japan, and the United States by integrating biochemical, clinical, nutritional, and regulatory perspectives to elucidate both therapeutic and preventive potentials.

## 2. Biochemical Metabolism of Medium-Chain Fatty Acids

### 2.1. Physicochemical Characteristics and Transport

MCTs consist of a glycerol backbone esterified with three medium-chain fatty acid (MCFA) molecules. Broadly defined, MCFAs are saturated fatty acids ranging from hexanoic acid (C6:0) to dodecanoic acid (C12:0) [8]. MCFAs are relatively abundant in coconut oil and palm kernel oil, both containing approximately 50% dodecanoic acid (C12:0) [9]. Commercially available MCTs, however, predominantly comprise octanoic acid (C8:0) and decanoic acid (C10:0), reflecting the origin of MCT production: utilizing low-melting-point, underutilized fatty acids from coconut oil [10,11]. At room temperature, MCTs are nearly colorless, transparent, low-viscosity liquids with no distinctive odor or taste [12].

Due to their shorter chain length, MCTs are digested and absorbed via pathways distinct from long-chain triglycerides (LCTs). Both MCTs and LCTs are initially hydrolyzed by lingual lipase, but MCTs are hydrolyzed 5–8 times faster than LCTs [12]. Additionally, MCTs are also efficiently hydrolyzed by gastric lipase [13,14]. Upon entering the small intestine, LCTs are hydrolyzed by bile and pancreatic lipase, form micelles, and are absorbed by epithelial cells. MCTs, conversely, exhibit lower dependence on bile and pancreatic lipase. In populations with immature secretion—such as premature infants or those with cholestasis—MCT-rich formulas can achieve higher absorption rates than LCT-based formulas [15,16,17]. Furthermore, MCTs do not markedly stimulate cholecystokinin (CCK) secretion, which triggers bile and pancreatic release, supporting their reduced dependence on these digestive components [18]. After absorption, LCTs are re-synthesized into triglycerides, incorporated into chylomicrons, secreted into lymphatics, and distributed systemically. In contrast, MCT-derived fatty acids—particularly C8:0 and C10:0—are largely transported as free fatty acids via the portal vein, whereas the contribution of lymphatic transport as chylomicrons can increase with chain length (especially toward C12:0) [18,19].

### 2.2. Mitochondrial β-Oxidation of MCFAs

MCFAs (particularly C8:0 and C10:0) reach the liver at high concentrations via the portal system and undergo rapid mitochondrial β-oxidation. The inner mitochondrial membrane exhibits low permeability to fatty acyl-CoA. Long-chain fatty acids (LCFAs) are converted to acylcarnitines via the carnitine shuttle, reconverted to acyl-CoA in the matrix, and then oxidized [19]. MCFAs, at least in the liver and kidney, exhibit low dependence on carnitine transport and undergo β-oxidation more directly [20]. However, carnitine dependence can be higher in extrahepatic tissues such as heart and skeletal muscle [20]. Indeed, CPT1 inhibition reduces LCFA oxidation but octanoate (C8:0) oxidation tends to be less sensitive to CPT1 inhibition [21]. Notably, C10 and C12 MCFAs resemble LCFAs more closely than C8, indicating a chain length-dependent gradient in metabolic properties [20].

Regarding metabolic regulation, CPT1 serves as the key control point for LCFA β-oxidation. Through CPT1 inhibition by malonyl-CoA (an ACC product), LCFA oxidation is suppressed in the fed state and disinhibited during fasting, coordinating fatty acid synthesis and oxidation [22,23]. Because MCFA oxidation is relatively less dependent on CPT1, it is less susceptible to malonyl-CoA regulation, allowing β-oxidation to commence rapidly, even postprandially. This may contribute, at least in part, to the rapid production of acetyl-CoA and subsequent increase in ketone body synthesis following MCT intake [24]. Consistently, human studies show that MCT intake increases postprandial ketone body production and energy metabolism compared to LCT intake [25]. The magnitude of ketone body elevation is chain length-dependent, with C8:0 exhibiting stronger ketogenic effects than C10:0 or C12:0 [26].

### 2.3. ω-Oxidation of MCFAs

**Figure 1:**
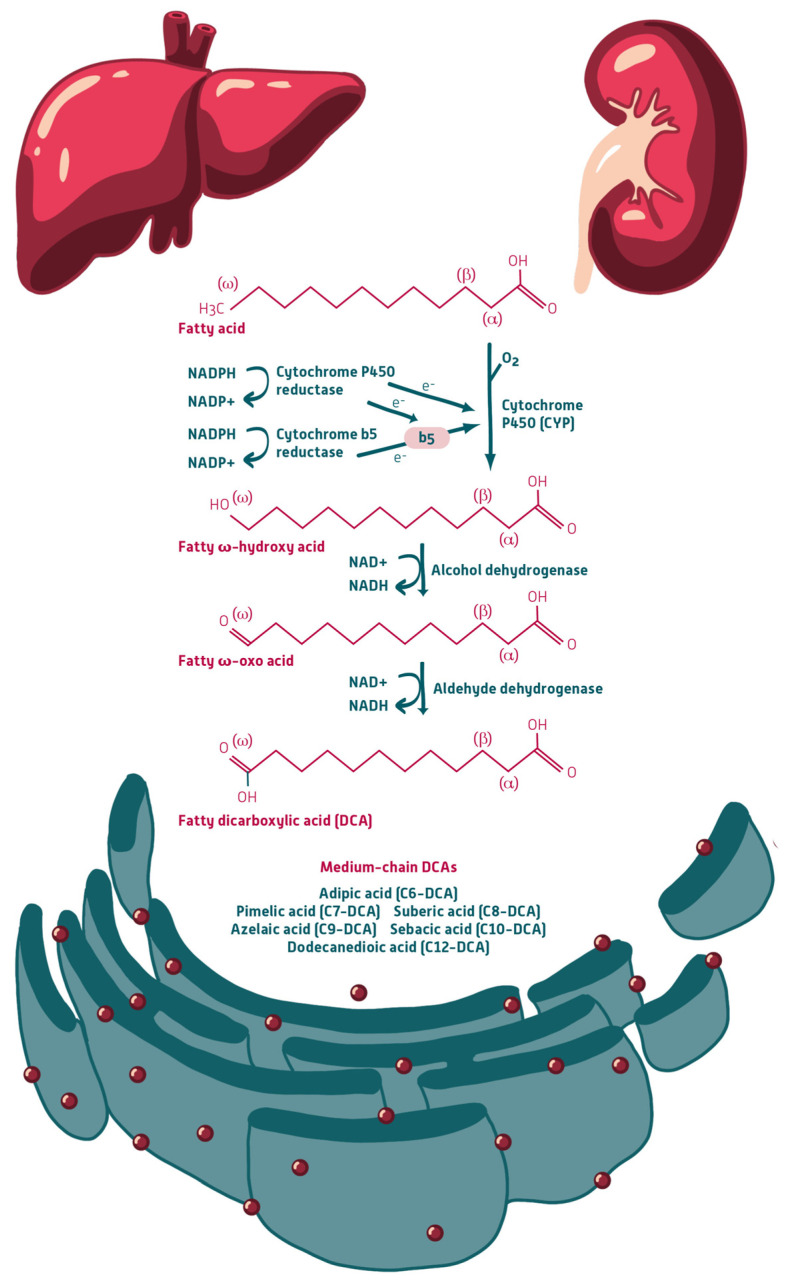
Enzymatic ω-oxidation to dodecanedioic acid (C12-DCA) illustrated by the example of lauric acid (C12). NADPH: Nicotinamide adenine dinucleotide phosphate (reduced form); NADP^+^: Nicotinamide adenine dinucleotide phosphate (oxidized form); NADH: Nicotinamide adenine dinucleotide (reduced form); NAD^+^: Nicotinamide adenine dinucleotide (oxidized form); ω (omega): Terminal carbon of the fatty acid chain; α (alpha): Carbon adjacent to the carboxyl group; DCA: Dicarboxylic acid.

In addition to mitochondrial β-oxidation, MCFAs can be metabolized via ω-oxidation, an auxiliary pathway that converts monocarboxylic fatty acids into dicarboxylic acids (DCAs). ω-Oxidation is initiated in the endoplasmic reticulum by NADPH-dependent hydroxylation of the terminal (ω) carbon, catalyzed by cytochrome P450 (CYP) enzymes of the CYP4A and CYP4F subfamilies [27]. The resulting ω-hydroxy fatty acids are sequentially oxidized by alcohol and aldehyde dehydrogenases to yield DCAs, which are subsequently activated and primarily degraded via peroxisomal β-oxidation [27]. MCFAs (C8–C12) are particularly relevant substrates for ω-oxidation, with enzyme affinity increasing with chain length, favoring C10 and C12 over C8 (Figure 1) [27].

While ω-oxidation contributes only marginally to total fatty acid oxidation under normal physiological conditions, its activity increases markedly when mitochondrial β-oxidation is impaired or saturated [28,29]. Such impairment may arise from carnitine deficiency, dysfunction of β-oxidation enzymes, or metabolic states associated with elevated lipid flux [2,27,30].

Under these conditions, fatty acids are increasingly metabolized via ω-oxidation, producing linear dicarboxylic acids such as adipic (C6-DCA), suberic (C8-DCA), and sebacic acids (C10-DCA) [2,30,31]. These metabolites accumulate and are excreted in the urine, serving as well-established biochemical markers of enhanced ω-oxidation [27,29,31]. Elevated urinary excretion of these DCAs is also observed during fasting, insulin resistance, diabetes, and low-carbohydrate intake, reflecting increased fatty acid flux and metabolic overflow [27,29].

Tissue specificity further modulates the relevance of ω-oxidation. The pathway is most active in liver and kidney, where it serves as a metabolic safeguard to limit fatty acid accumulation when mitochondrial β-oxidation capacity is exceeded [27]. In contrast, tissues such as heart and skeletal muscle exhibit minimal ω-oxidation activity, and DCA β-oxidation contributes negligibly to their overall energy metabolism [27].

In the context of MCFAs, rapid hepatic uptake and oxidation—particularly following MCT intake—may transiently overwhelm mitochondrial β-oxidation capacity, thereby enhancing ω-oxidation and downstream DCA production, including sebacic acid from decanoic acid [27]. Thus, ω-oxidation complements mitochondrial β-oxidation of MCFAs and becomes metabolically and diagnostically relevant under conditions of mitochondrial dysfunction or increased lipid load [3,27,32].

## 3. Industrial Synthesis and Production of MCTs

MCTs are primarily composed of caprylic (C8) and capric (C10) fatty acids which are esterified with glycerol. Compared with long-chain triglycerides, MCTs are liquid at room temperature, exhibit lower melting points, smaller molecular volumes, and partial water solubility, allowing stable emulsions at low concentrations [12,33,34,35]. These properties confer multiple functional advantages, including high solvency for lipophilic compounds, low viscosity, heat stability, and a neutral organoleptic profile, making MCTs suitable for food, pharmaceutical, and cosmetic applications [35].

Natural oils contain MCTs only in combination with long-chain fatty acids; therefore, industrial production of pure MCTs requires dedicated synthesis [35,36]. Coconut oil and palm kernel oil are the primary feedstocks due to their high medium-chain fatty acid content, renewable origin, and high yield [35,36,37].

Industrial production generally begins with hydrolysis of the raw oils to release free fatty acids and glycerol. The fatty acids are fractionated according to chain length to obtain a lower-boiling fraction enriched in C8 and C10 fatty acids. These can then be directly esterified with glycerol via chemical methods at high temperatures (≥160 °C), with continuous water removal to drive the reaction to completion [38]. Residual free acids are commonly removed by vacuum distillation, and the crude MCTs are deodorized to eliminate volatiles.

Alternatively, triglycerides can be converted to glycerol and fatty acid methyl esters using basic methanolates in a stoichiometric fashion. The methyl esters are separated by fractional distillation and further processed either by direct esterification with glycerol or by hydrolysis to obtain pure fatty acids and methanol. From an industrial perspective, solvent-free processes are preferred, as they reduce cost and processing time [38,39].

Enzymatic synthesis provides a milder and more selective approach [38]. For example, immobilized lipases, attached to anionic exchange resins, catalyze esterification or interesterification of pure glycerol and medium-chain fatty acids, albeit with very long reaction times [38].

Heydinger-Galante, J. et al. described a conventional process in which a mixture of caprylic and capric acids with glycerol was circulated through a packed column containing immobilized *Rhizomucor miehei*, with continuous water removal [40]. After several days, the triglyceride content reached 93%. To improve industrial feasibility, a two-step heating process was implemented, achieving the same yield in only 17 h [40].

Schoerken, U. et al. outlined a process coupling enzymatic esterification with selective back-hydrolysis and distillation [41]. Caprylic acid, glycerol, and immobilized *Candida antarctica B* lipase in the presence of sodium carbonate were incubated at 60 °C. After filtration of the immobilized enzyme, liquid lipase was added, and the mixture held at room temperature. After to the pH-adjustment caprylic acid and monoglycerides were removed by distillation. Subsequent treatment with sodium hydroxide produced MCTs with very high triglyceride content and low hydroxyl values, yielding light, fast-spreading, and non-greasy products suitable for cosmetic applications [41].

Dragan, V. described a transesterification method using methyl caprate and methyl caprylate with glycerol, catalyzed by immobilized Lipozyme^®^ 435 with silica gel addition at 65 °C under nitrogen [42]. The process achieved approx. 42% glycerol esterification after 5.5 h, significantly improving reaction rates compared to systems without silica gel [42].

Rongione, J.C. and Heydinger-Galante, J. developed a method to minimize monochloropropanediol (MCPD) and glycidyl ester contaminants by converting chlorinated by-products into non-chlorinated esters using the respective carboxylates [43]. For C8/C10 fatty acids, sodium carbonate and glycerol were reacted at temperatures above 245 °C, effectively reducing total 3-MCPD to <0.15 mg/kg [43]. The outlined technology improves MCT quality by chemically converting and removing harmful contaminants during or after processing, yielding a cleaner, safer, and higher-purity MCT product with improved regulatory and food-safety profiles.

Solvent-free synthesis of pure tricaprylin (C8), tricaprin (C10), and trilaurin (C12) using metal oxide catalysts has also been reported, albeit on a small scale [36]. Alternative feedstocks include microalgae (*Symbiodinium microadriaticum*) grown photoautotrophically, and genetically engineered oilseed crops expressing β-ketoacyl-ACP synthase from *Cocos nucifera*, enabling production of medium-chain fatty acids or triglycerides in plants [37,44].

Salvador and Apostol describe a process for producing MCTs with a substantial amount of lauric acid (C12, ≥5%) using methyl esters from fractionated coconut or palm kernel oil. Acidic, alkaline, or metal oxide catalysts with continuous methanol removal enabled high-purity MCT synthesis [38].

Recent patents by Lochmann, Reyer, and Stehr introduced mild synthetic pathways combining enzymatic esterification, protected glycerol (solketal), and post-synthesis purification using bisulfite to produce MCTs with triglyceride content greater than 99.5%, acid and hydroxyl values ≤0.1 mg KOH/g, and toxic impurities below 0.1 ppm [45,46]. This approach improves quality and safety by reducing process-related contaminants such as 3-MCPD and glycidyl ester precursors during production, thereby enhancing regulatory compliance and food-safety acceptance while maintaining a high-purity MCT composition suitable for food and nutrition applications. In a similar approach, the same authors present mild synthetic pathways for producing high-purity tricaprylin (glycerol trioctanoate). Using enzyme-catalyzed esterification—such as employing *Candida antarctica* lipase—and direct conversions with caprylic acid or its anhydride, the described processes also include routes using solketal for controlled esterification, as well as hydrogenation of unsaturated C8-triglycerides. These methods ensure minimal impurity formation and high yields [46].

## 4. Application of Medium-Chain Fatty Acids (MCTs)

The following sections summarize historical, clinical, and regulatory perspectives on the use of medium-chain triglycerides in Germany, Japan, and the United States.

### 4.1. Insights from Germany

Owing to their distinct metabolic characteristics and rapid intestinal absorption, MCTs and their constituent MCFAs were introduced in the 1960s as a dietary energy source for clinical nutrition. In Germany, they were subsequently marketed as foods for special medical purposes (FSMP) for the dietary management of gastroenterological and hepatological disorders. Over time, their clinical use has expanded to additional indications, including neurological disorders managed with ketogenic diets and inherited metabolic diseases such as defects in long-chain fatty acid β-oxidation [5].

#### 4.1.1. Gastrointestinal and Liver Disorders

The clinical rationale for the use of MCTs in gastrointestinal and liver diseases is primarily based on their simplified digestion and absorption compared with long-chain triglycerides. In contrast to long-chain fatty acids, medium-chain fatty acids do not require bile salt-mediated micelle formation for absorption and are transported predominantly via the portal venous system rather than the lymphatic circulation. Table 1 summarizes the physiological rationale for the use of MCTs in selected gastrointestinal and liver disorders.

#### 4.1.2. Ketogenic Diet (KD)

Ketogenic diets (KDs) are high-fat, carbohydrate-restricted dietary regimens that induce nutritional ketosis, a metabolic state in which hepatic fatty acid oxidation generates ketone bodies, primarily acetoacetate and β-hydroxybutyrate, that serve as alternative energy substrates for the brain and partially mimic fasting metabolism [52]. Since their introduction in the 1920s, KDs have been used as medically supervised therapies for drug-resistant epilepsy and are recommended as first-line treatment for inherited disorders of cerebral energy metabolism, including glucose transporter type 1 (GLUT1) deficiency and pyruvate dehydrogenase complex deficiency (PDHD) [53].

Several dietary protocols have been developed to improve tolerability and adherence to ketogenic therapy, including the classical ketogenic diet (CKD), the modified Atkins diet, the low glycemic index treatment and the medium-chain triglyceride (MCT) ketogenic diet [53]. The MCT ketogenic diet, introduced by Huttenlocher et al. in 1971, exploits the higher ketogenic efficiency of medium-chain triglycerides compared with long-chain triglycerides (LCTs) [54,55]. Because MCTs generate more ketone bodies per unit of energy, their inclusion permits a higher intake of carbohydrates and protein while maintaining therapeutic ketosis [56]. Clinical studies have shown that the MCT ketogenic diet is comparably effective to the classical ketogenic diet for seizure control [57].

The anticonvulsant effects of ketogenic diets have traditionally been attributed to ketone body production [58]. However, the degree of ketosis does not consistently correlate with seizure reduction, suggesting that additional mechanisms contribute to therapeutic efficacy [59]. This observation has prompted increasing interest in the role of MCFAs, particularly decanoic acid (C10:0) [6]. Decanoic acid readily crosses the blood–brain barrier and has been shown to reduce excitatory neurotransmission and neuronal network excitability in vitro, increase seizure threshold in animal models, and enhance mitochondrial biogenesis as well as respiratory chain and catalase activity [6,60,61,62]. These effects may be potentiated by octanoic acid (C8:0), which appears to spare decanoic acid from oxidative metabolism in neuronal-like cells [63].

MCTs exert anticonvulsant effects through both indirect and direct mechanisms: indirectly by sustaining ketone body production and thereby supporting cerebral energy metabolism, and directly through decanoic acid-mediated inhibition of excitatory AMPA receptors [6,62,64,65].

#### 4.1.3. Long-Chain Fatty Acid Oxidation Disorders (LC-FAODs)

LC-FAODs are rare, autosomal recessive inborn errors of metabolism characterized by impaired mitochondrial β-oxidation of long-chain fatty acids. They include disorders of the carnitine cycle—carnitine palmitoyltransferase I and II deficiencies (CPT-I and CPT-II) and carnitine–acylcarnitine translocase deficiency (CACT)—as well as defects in the mitochondrial β-oxidation pathway, such as very-long-chain acyl-CoA dehydrogenase deficiency (VLCAD) and long-chain 3-hydroxyacyl-CoA dehydrogenase/mitochondrial trifunctional protein deficiency (LCHAD/MTP). These disorders lead to acute energy crises and chronic energy deficiency [66]. Clinical manifestations include fasting- or illness-induced hypoketotic hypoglycemia, hepatic dysfunction with hyperammonemia, cardiomyopathy, and exercise-induced rhabdomyolysis [66,67].

Dietary therapy requires restriction of long-chain fatty acids, as their mitochondrial transport or β-oxidation is defective depending on the specific enzyme deficiency [67,68]. MCT supplementation is therefore an important source of alternative energy because medium-chain fatty acids can enter mitochondria independently of the carnitine transport system and bypass the impaired metabolic steps [67].

In addition, pre-exercise administration of MCT oil (0.25–0.5 g/kg body weight) approximately 20 min before strenuous physical activity has been shown to reduce intramyocellular accumulation of long-chain fatty acids and to lower steady-state heart rate, indicating improved metabolic efficiency during exercise following MCT supplementation [67,69,70].

In contrast, in medium-chain fatty acid oxidation disorders such as medium-chain acyl-CoA dehydrogenase (MCAD) deficiency, MCT supplementation is generally not indicated [71].

#### 4.1.4. Further Applications

Beyond the established clinical indications described above, MCTs have also been investigated in other contexts, including weight reduction, obesity management, and sports nutrition [72,73]. Due to their rapid oxidation and potential effects on thermogenesis and satiety, MCTs have been proposed to contribute to body weight regulation by increasing energy expenditure, reducing food intake, and limiting fat deposition in adipose tissue. The German Nutrition Society (Deutsche Gesellschaft für Ernährung, DGE) has critically evaluated medium-chain triglycerides (MCTs) in its guideline on dietary fat intake and the prevention of selected nutrition-related diseases [61]. Short-term clinical studies suggest that partial replacement of conventional dietary fats with MCTs may lead to modest reductions in body weight and adipose tissue mass [61]. However, evidence regarding the long-term efficacy of substituting long-chain triglycerides (LCTs) with MCTs for obesity management remains insufficient [61]. Consequently, MCTs cannot currently be recommended as a reliable or effective strategy for sustained weight reduction [61].

MCTs have also been investigated as potential ergogenic aids in sports nutrition [7]. This hypothesis is based on their rapid intestinal absorption and oxidation, which could theoretically provide a readily available energy substrate during exercise and spare muscle glycogen [7,74]. However, controlled studies have not demonstrated consistent improvements in muscle glycogen utilization, endurance capacity, or overall exercise performance following MCT supplementation [7,75]. Consistent with these findings, the position statement of the DGE working group on sports nutrition concludes that MCT supplementation does not enhance athletic performance [73]. Accordingly, although MCTs have been proposed as a rapidly available energy substrate during exercise, current evidence does not support a consistent ergogenic benefit [73].

### 4.2. Insights from Japan

Japan is one of the most rapidly aging countries in the world and is consequently facing a growing prevalence of age-related health issues, including malnutrition, frailty, and sarcopenia. These conditions are interrelated and represent major risk factors for dependency on long-term care [76]. Therefore, their prevention and management are of paramount importance for extending healthy life expectancy. Unlike LCTs, MCTs possess a unique metabolic pathway that allows for their rapid utilization as an energy source. Therefore, MCTs have potential applications in addressing the nutritional challenges faced by the elderly population in Japan.

Malnutrition is a condition characterized by a deficiency in energy and protein, leading to an insufficient supply of nutrients required to maintain healthy bodily functions. A collaborative study across three East Asian countries, including Japan, reported a markedly high prevalence of malnutrition among adults aged 70 and older [77]. Since MCTs are lipids with a high energy density per unit weight, MCTs are more rapidly metabolized for energy than LCTs, thereby imposing a lower physiological burden on elderly individuals, who often have diminished digestive function. Furthermore, MCTs are tasteless and odorless, which offers the practical advantage of easy incorporation into various foods and seamless integration into the daily diet. These properties establish MCTs as a valuable nutritional ingredient for combating malnutrition.

The role of MCTs extends beyond mere energy supplementation. An intervention study in elderly Japanese individuals at risk of malnutrition demonstrated that continuous daily intake of 6 g of MCTs significantly improved serum prealbumin and albumin levels compared to intake of 6 g of LCTs [78]. Although definitive clinical evidence is not yet available, findings from in vitro and animal studies suggest that the underlying mechanism may involve the activation of protein synthesis pathways. A study in malnourished rats showed that MCTs ingestion increased plasma insulin concentrations and activated the Akt/mTOR pathway, a key signaling cascade for protein synthesis [79]. It has also been suggested that C10 fatty acids, in particular, may enhance GLP-1 secretion via the G protein-coupled receptor 84 (GPR84) in the small intestine, subsequently increasing blood insulin levels [80]. These findings suggest that MCTs have the potential to suppress protein catabolism and promote anabolism. However, it is crucial to note that although serum albumin has been traditionally used as a marker for nutritional status, it is also strongly associated with inflammation and may not exclusively reflect nutritional state [81]. More recently, the Global Leadership Initiative on Malnutrition (GLIM) criteria has been proposed as an international consensus for the diagnosis of malnutrition, combining phenotypic criteria (weight loss, low BMI, reduced muscle mass) and etiologic criteria (reduced food intake/absorption, inflammation) [82,83]. One of the phenotypic criteria, reduced muscle mass, is intrinsically linked to frailty and sarcopenia. Consequently, MCTs may have clinical applications as a dietary component that could contribute to improvements in muscle mass.

A combined data analysis of two clinical trials involving frail elderly Japanese participants reported that a 3-month intervention with 6 g of MCTs per day resulted in significant improvements in body weight, arm muscle area, calf circumference, handgrip strength, knee extension time, and walking speed compared to LCTs intake [84]. On the other hand, it is important to note that the direct effects of MCTs on muscle mass per se have yielded inconsistent results. A 12-week intervention study in healthy elderly Japanese individuals found that a relatively low daily dose of 2 g of MCTs led to a significant improvement in knee extension strength compared to an LCTs control group, but no corresponding increase in muscle mass was observed [85]. Furthermore, a retrospective cohort study of 1080 post-stroke Japanese patients indicated that MCTs intake alone did not improve skeletal muscle mass index (SMI), suggesting a synergistic association with resistance exercise [86]. These results imply that MCTs may exert their beneficial effects on muscle function more potently when combined with exercise, rather than by directly increasing muscle mass. Further well-designed studies are required to clarify long-term effects on muscle mass and physical function and to define optimal dosing strategies, timing of intake, and the relative contributions of individual medium-chain fatty acids (C8 and C10) in different clinical settings.

The characteristics of MCTs as a rapidly available energy source have long garnered attention in the field of sports nutrition, particularly for endurance athletes. However, earlier studies investigating the acute intake of MCTs before exercise reported limited or inconsistent effects on performance [87,88].

In recent years, however, two intervention studies have been conducted on physically active Japanese individuals to examine the effects of daily MCTs ingestion (6 g/day) on endurance performance. Both studies reported that ingestion of MCTs significantly enhanced endurance performance by promoting fat utilization as an energy substrate [89,90]. This enhancement of fat utilization following continuous ingestion of MCTs has also been observed in several intervention studies involving sedentary healthy Japanese individuals, suggesting it is a fundamental physiological effect of MCTs [91,92].

The primary mechanism for the enhanced endurance performance in these studies is considered to be muscle glycogen sparing, resulting from increased fat utilization. Findings from an animal study by Fukazawa et al. provide insight into the potential molecular mechanisms underlying this metabolic adaptation [93]. Their study reported that while rats fed a high-fat diet composed solely of LCTs showed a marked increase in the expression of pyruvate dehydrogenase kinase 4 (PDK4), enzyme that suppresses glucose utilization, in skeletal muscle, rats fed an MCT-containing high-fat diet did not show this increase. Instead, the latter group exhibited enhanced ketone body utilization in skeletal muscle.

This finding suggests that long-term intake of MCTs induces a metabolic adaptation that allows for the efficient utilization of fat without compromising the glycolytic capacity required for high-intensity exercise. This indicates that continuous MCTs ingestion has the potential to create an ideal metabolic state for endurance athletes. Future studies in this field are highly anticipated.

### 4.3. Insights in the United States

In the late 1950s in the United States (U.S.), interest in potential therapeutic uses of MCTs led to metabolic studies demonstrating that MCFAs are absorbed and metabolized differently from LCFAs, undergoing rapid intestinal absorption and direct transport to the liver via the portal circulation. Thereafter, clinical trials investigated potential clinical applications of medium-chain triglycerides for disorders of fat absorption. A decade of MCT oil research was summarized in a monograph edited by Jesse R. Senior, which also included contributions from investigators such as Sami A. Hashim, Theodore B. VanItallie, Norton J. Greenberger, and Thomas G. Skillman [94]. A classic study by Bergen et al. further demonstrated that MCFAs are readily oxidized and converted to ketone bodies [94]. Thereafter, MCT-containing nutritional enteral formulations were used in hospitals for conditions such as pancreatic insufficiency, cystic fibrosis, short-bowel syndrome, and severe malnutrition. In addition, intravenous lipid emulsions containing MCT/LCT mixtures (often 50:50) were studied in clinical trials for people who were unable to tolerate enteral feeding [95,96]. However, most intravenous emulsions currently used in the U.S. contain soybean oil-based long-chain triglycerides apart from several U.S. Food and Drug Administration (FDA)-approved products that contain MCTs as part of a mixed lipid formulation.

In the 1970s in the U.S., clinical trials by Tantibhedhyangkul and Hashim documented the physiological and therapeutic effects of MCTs in alleviating steatorrhea in premature infants [97]. Subsequently, MCT oil supplementation of enteral feedings became widely adopted in neonatal intensive care units for extremely premature infants. Later studies by Jensen, Buist, and Wilson demonstrated that very small infants absorb medium-chain fatty acids from MCTs more efficiently than long-chain fatty acids, supporting the use of MCT-containing formulas to improve fat absorption in premature infants [98]. By the early 1980s in the U.S., MCT oil was incorporated into specialized formulas for premature infants with immature fat digestion, with MCTs typically providing 30 to 50% of total fat to enhance lipid absorption. Human milk naturally contains MCFAs [99,100]. Consequently, since the early 1980s, major U.S. infant formula manufacturers, whose products are now marketed worldwide, have included coconut oil or palm kernel oil in formula fat blends designed to approximate the fatty acid composition of human milk by supplying predominantly C12, along with smaller amounts of C8 and C10 fatty acids.

Meanwhile, the development of the MCT ketogenic diet in the U.S. in the early 1970s as an alternative to the classic ketogenic diet expanded the use of MCTs in dietary therapies for drug-resistant epilepsy. In pioneering work, Huttenlocher and colleagues demonstrated that diets enriched with medium-chain triglycerides could produce sustained ketosis while permitting greater carbohydrate and protein intake than the classical ketogenic diet, and that this MCT-based regimen reduced seizure frequency in many patients with refractory epilepsy [54,55].

More recently, research has explored the potential role of MCT-derived ketones as alternative metabolic substrates to glucose in conditions associated with altered brain energy metabolism and memory impairment. For example, in a randomized controlled trial, Fortier et al. reported that daily consumption of a ketogenic MCT drink increased circulating ketone levels, enhanced brain ketone uptake measured by PET imaging, and improved some cognitive measures in individuals with mild cognitive impairment [101]. As another example, Taylor et al. conducted a 3-month pilot study of an MCT oil supplemented ketogenic diet in individuals with Alzheimer’s disease. Participants who completed the intervention achieved nutritional ketosis and demonstrated a significant improvement in ADAS-cog scores that reverted to baseline after a washout period, indicating that ketogenic metabolic therapy may transiently improve cognitive performance in mild Alzheimer’s disease [102].

Collectively, these developments illustrate how early metabolic studies in the United States laid the foundation for diverse clinical and nutritional applications of MCTs, ranging from the management of malabsorption and neonatal nutrition to ketogenic dietary therapies and emerging metabolic approaches for neurological disorders.

### 4.4. Practical Considerations for Using MCTs

Several points should be considered for the nutritional application of MCTs. Ingestion of a large quantity of MCTs at once can cause gastrointestinal symptoms such as diarrhea and abdominal pain [7]. To enhance tolerability, a gradual introduction with progressive dose increases is generally recommended.

#### 4.4.1. Clinical Practice in Germany

In German clinical nutrition practice, MCT supplementation is typically introduced in a structured, stepwise manner. For target daily intakes of approximately 15 g, supplementation commonly begins at low doses (e.g., 5 g/day), with gradual dose increases guided by individual gastrointestinal tolerance [5]. In adult patients with higher intended intakes (≥20 g/day), supplementation may be initiated at higher starting doses and increased incrementally, provided tolerability is maintained [5]. To minimize gastrointestinal side effects, the total daily MCT intake is generally divided across several meals (three to five per day). Dosing is individualized, with body weight frequently used as a reference parameter, corresponding to approximately 1 g MCT per kg body weight per day. For practical dose estimation, 3 g of MCT approximates one teaspoon, and 6 g corresponds to one tablespoon of MCT oil [5]. Incorporation of MCTs into foods rather than consumption as a pure oil is recommended; thorough mixing into semi-solid or liquid food matrices may improve dispersion and reduce gastrointestinal intolerance. During prolonged MCT supplementation (>3 weeks), an adequate intake of essential fatty acids must be ensured [5].

#### 4.4.2. Practice in Japan

In Japan, MCTs are widely used not only as oil-based products but also in powdered formulations that are readily dispersible in beverages and foods. These formulations expand practical options for daily intake and may facilitate long-term adherence by accommodating individual preferences and consumption contexts. As in German practice, gradual dose increases are generally advised to minimize gastrointestinal adverse effects, although the dosages applied in clinical and nutritional studies vary considerably. This heterogeneity in dosing and formulation may partly contribute to the variability in reported clinical outcomes.

#### 4.4.3. Clinical Practice in the United States

MCTs have become widely incorporated into clinical nutrition practice in the U.S. In clinical settings, MCTs are commonly used as part of nutritional management strategies for disorders involving impaired fat digestion, altered energy metabolism, or increased energy requirements.

Dietitians frequently incorporate MCT oil into enteral feeding regimens or specialized diets because MCFAs are absorbed efficiently even in the presence of pancreatic insufficiency or bile acid deficiency. MCT supplementation is also widely used in ketogenic dietary therapies, where it helps sustain the production of ketone bodies while allowing a less restrictive macronutrient composition compared with classical ketogenic diets. Guidelines for ketogenic dietary therapy summarized by Kossoff and colleagues on behalf of the International Ketogenic Diet Study Group emphasize careful titration of MCT intake to optimize ketosis while minimizing gastrointestinal intolerance [53].

In clinical settings in the U.S. as in Germany and Japan, MCT oil is typically introduced gradually and divided across several meals daily to improve tolerance.

## 5. Regulatory Frameworks and Innovation Policies

### 5.1. The Case of MCTs in Germany

MCTs have been available in Germany since the 1960s and were initially introduced for the dietary management of patients with metabolic or digestive impairments [103]. This early regulatory recognition facilitated their clinical use in conditions such as fat malabsorption, exocrine pancreatic insufficiency, cholestatic liver diseases, and disorders of bile acid metabolism, as well as in ketogenic diets for neurological conditions and in long-chain fatty acid oxidation disorders (LC-FAODs)

Over time, the regulatory framework evolved from national regulations, such as the German Regulation on Dietetic Foods, to European legislation. The current legal basis is primarily defined by Regulation (EU) No 609/2013 and Commission Delegated Regulation (EU) 2016/128, which established the category of Foods for Special Medical Purposes (FSMPs), replacing earlier dietary classifications. In Germany, these EU provisions are complemented by the Regulation on Foods for Specific Groups (Lebensmittel für bestimmte Verbrauchergruppen-Verordnung, LMBVV).

Under this framework, MCTs are classified as FSMPs if they meet strict criteria: they must address disease-related nutritional requirements under medical supervision and serve a dietary management function rather than a drug-based function. Typically, MCTs are nutritionally incomplete FSMPs intended to supplement—but not replace—normal nutrition.

Currently, FSMPs enter the market without prior governmental review or demonstration of efficacy. Manufacturers must notify the Federal Office of Consumer Protection and Food Safety (BVL) on the first day of commercialization, submitting a label sample. Notifications are forwarded to the relevant state authorities, which conduct market surveillance and sample-based inspections.

The European Food Safety Authority (EFSA) published in 2015 its Scientific and Technical Guidance on Foods for Special Medical Purposes in the context of Article 3 of Regulation (EU) No 609/2013. This guidance specifies the type and format of information and scientific data that manufacturers must, upon request, provide in order to demonstrate whether a product qualifies as an FSMP [104]. The dossier covers product characterization, intended use, target patient population, disease context, dietary management role, and conditions for medical supervision [104].

**Figure 2:**
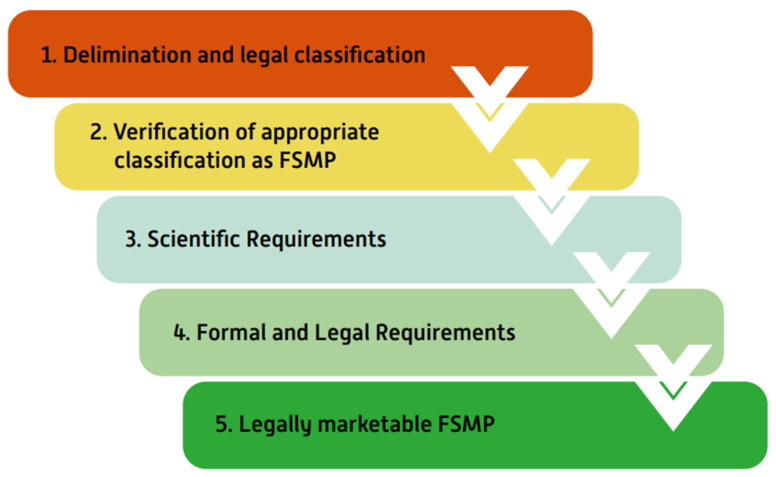
Federal Office of Consumer Protection and Food Safety (BVL) Assessment Scheme for the Characterization of Food for Special Medical Purposes (FSMP) [105]; FSMP: Food for Special Medical Purposes.

In Germany, this EU framework is complemented by national guidance: the Federal Office of Consumer Protection and Food Safety (BVL) and the Federal Institute for Drugs and Medical Devices (BfArM) issued a second edition position paper providing a structured framework and decision tree to support consistent FSMP classification, offering orientation for manufacturers and state authorities and promoting uniform nationwide regulatory practice (Figure 2) [105].

Together, EFSA guidance and the BVL/BfArM position paper provide regulatory support that enables predictable, scientifically substantiated development of FSMPs, including novel formulations, delivery formats, and combination products, while ensuring patient safety.

### 5.2. The Case of MCTs in Japan

In Japan, foods are broadly categorized into two types: Foods in General, which cannot label functional claims, and Foods with Health Claims, which can label functional claims. Foods with Health Claims comprise three types: (i) Foods with Nutrient Function Claims, (ii) Foods for Specified Health Uses (FOSHU), and (iii) Foods with Function Claims [106]. MCT products are distributed as not only Foods in General but also (ii) FOSHU and (iii) Foods with Function Claims.

(i) Foods with Nutrient Function Claims can be used to supplement or complement daily requirement of nutrients which tend to be insufficient in everyday diet. Given that the food product contains certain amounts of nutrient whose function has already been substantiated by scientific evidence, it can bear a nutrient function claim prescribed by the standards without submitting a notification to the government. The nutrients whose function has already been substantiated by scientific evidence are limited to omega-3 fatty acids, 6 minerals, and 13 vitamins; MCFAs are not included [107].
(ii) FOSHU are scientifically recognized as helpful for maintaining and promoting health and are permitted to bear claims such as “Slows cholesterol absorption.” The government evaluates the claimed effects and safety, and the Secretary-General of the Consumer Affairs Agency gives approval for the labeling of each food product that satisfies the requirements. The system was established in 1991. Since then, 1034 items have been approved and 6 products contain MCFAs. An example of approved labeling for an edible cooking oil states the following: “This oil contains MCFAs and restrains the accumulation of body fat. This oil is recommended for people beginning to be concerned about body fat and obesity; it should be used in place of the usual edible oil.” [108]. As supporting evidence, studies comparing the effects on body fat accumulation between a diet containing 14 g of medium- and long-chain triacylglycerols (MLCTs) and a diet using regular cooking oil showed that, in subjects with an average BMI of 24.6 ± 0.4 kg/m^2^, body fat mass, waist circumference, hip circumference, subcutaneous fat area, and visceral fat area were significantly reduced after 4 to 12 weeks of consumption compared to the control oil group [109].
(iii) Foods with Function Claims can be labeled with function claims based on scientific evidence, under the food business operator’s own responsibility. Functional claims based on scientific evidence indicate that the functional substances contribute to specific health purposes (excluding those related to disease risk reduction) by maintaining or enhancing health. Information on the evidence supporting the safety and effectiveness of the product are submitted to the Secretary-General of the Consumer Affairs Agency before the product is marketed. However, unlike FOSHU, the product is not individually pre-approved by the Secretary-General of the Consumer Affairs Agency [110]. The system was established in 2015, and since then, 10,297 items have been notified. There are 93 items using MCFAs as the functional substances, and the functional claims are listed in Table 2. All notifications used systematic literature reviews as their scientific basis [111]. The studies included in the systematic literature reviews are listed in Table 3.

Both FOSHU and Foods with Function Claims are fundamentally intended for “healthy individuals.” While FOSHU may also target individuals “at risk of developing lifestyle-related diseases caused by dietary habits, etc.” [85], they cannot target patients, unlike FSMPs in Germany. FOSHU and Foods with Function Claims are distributed like Foods in General and are positioned as foods expected to achieve specific health benefits in healthy individuals. In Japan, MCT is widely anticipated to maintain and enhance the health of healthy individuals, not only patients.

### 5.3. Critical Appraisal of Evidence Supporting Japanese Functional Claims

The evidence supporting the FOSHU approval was based on intervention periods of 12 weeks, and the evidence supporting Foods with Function Claims involved intervention periods ranging from 2 to 12 weeks (Table 3). To evaluate long-term efficacy, studies with longer intervention durations are necessary. Also, the MCT oil used in the clinical trials in the above evidence was mainly composed of C8 and C10. It is not clear how C8 and C10 function, respectively, for each phenotype, and further research would be desirable. Moreover, given that ethnicity and dietary culture may influence an individual’s response to MCTs, it is important to conduct intervention trials with appropriately selected subjects in Germany to apply evidence obtained in Japan to the German population.

### 5.4. The Case of MCTs in the United States

In contrast to the product-specific functional food approval or notification systems used in some countries, the regulatory environment in the U.S. has primarily allowed the use of MCTs through ingredient-based safety determinations and specialized nutrition categories rather than through product-specific approvals. MCT oil is regulated as a conventional food ingredient that is generally recognized as safe (GRAS) under the Federal Food, Drug, and Cosmetic Act. MCTs entered the U.S. GRAS notification inventory relatively late compared with many traditional food ingredients because they were already widely used in clinical nutrition and infant formulas before the notification system was established.

The concept of “Generally Recognized as Safe” (GRAS) originated with the Food Additives Amendment of 1958 to the U.S. Federal Food, Drug, and Cosmetic Act [119]. This legislation established a formal regulatory framework requiring pre-market approval for new food additives while exempting substances that were already widely accepted as safe by qualified experts or substances that had a long history of use in food. Shortly after passage of the amendment, the U.S. Food and Drug Administration (FDA) published the first GRAS list in the Federal Register on December 9, 1958, identifying substances considered generally recognized as safe under specified conditions of use, which includes MCTs [120].

During the following decades, the FDA developed additional procedures to clarify and update GRAS determinations. In 1972, the agency established a GRAS affirmation petition process allowing manufacturers to request formal FDA confirmation of GRAS status. In 1997, the FDA replaced this process with the current voluntary GRAS notification program, under which manufacturers submit evidence supporting their own GRAS determination and the FDA responds with either a “no questions” letter or other comments [121]. Under this framework, ingredients that meet the GRAS standard, which is defined as a reasonable certainty of no harm under the intended conditions of use and general recognition of safety among qualified experts, may be used in foods without undergoing the full food-additive approval process.

Multiple manufacturers have submitted GRAS notifications to the U.S. Food and Drug Administration regarding the use of MCTs in foods and nutritional products. For example, GRAS Notice No. 449 addressed the use of MCTs in a variety of food categories, and the FDA responded with a “no questions” letter regarding the notifier’s GRAS conclusion [122]. Similarly, GRAS Notice No. 1049 addressed the use of MCTs in infant formula products [123].

In addition to their use as conventional food ingredients, MCTs have also been incorporated into dietary supplements and medical foods. In the U.S., MCT oil may be marketed as a dietary supplement if it complies with the requirements of the Dietary Supplement Health and Education Act, which was established in 1994, including ingredient safety, current good manufacturing practices, appropriate labeling, and restrictions on disease-related claims [124,125,126]. Since MCTs were marketed as an ingredient before 1994, they do not require pre-market FDA notification as a New Dietary Ingredient (NDI). Dietary supplements may make structure and functions claims, such as “supports energy metabolism” or “supports ketogenic metabolism”, but they cannot claim to diagnose, treat, cure, or prevent disease.

The medical food regulatory category was formally established in 1988 when the U.S. Congress added a statutory definition of medical foods to the Federal Food, Drug, and Cosmetic Act [127]. A medical food is defined in U.S. law as a food formulated to be consumed or administered enterally under the supervision of a physician for the specific dietary management of a disease or condition with distinctive nutritional requirements established by medical evaluation [128]. Like dietary supplements, a medical food cannot make drug-like claims to diagnose, treat, cure, or prevent disease.

Under the medical food framework, enteral formulations containing medium-chain triglycerides (MCTs) are used in the dietary management of conditions such as fat malabsorption, pancreatic insufficiency, and certain metabolic disorders. These formulations may include peptide-based enteral nutrition products or ketogenic diets used in the management of drug-resistant epilepsy. Single-ingredient MCT oil medical foods are less common but may provide energy substrates that bypass impaired long-chain fatty-acid oxidation pathways.

In the United States, MCT oil can be marketed as a conventional food ingredient, dietary supplement, component of infant formulas and clinical nutrition products, or medical food under the ingredient-based GRAS regulatory framework. In contrast, Germany and Japan regulate comparable products through different policy mechanisms, including medical nutrition regulations and functional food approval systems.

Viewed alongside the regulatory approaches in Germany and Japan, the U.S. framework illustrates how MCT-containing foods can be incorporated into national food systems through differing policy mechanisms, including ingredient-based safety determinations, medical nutrition regulation, and functional food approval systems.

### 5.5. Regulatory Comparison of MCTs in Germany and Japan

Unlike the United States, where the use of MCTs is allowed at the ingredient level, both Germany and Japan require product-specific approval or notification to label health function claims related to MCTs. However, The regulatory and innovation frameworks for MCTs differ markedly between these two countries. In Germany, MCTs are regulated as clinically targeted FSMPs to address disease-specific nutritional needs under medical supervision. In contrast, in Japan, MCTs are primarily positioned as Foods with Health Claims, designed to maintain and promote health in generally healthy populations or at-risk populations of lifestyle-related diseases (Table 4).

## 6. Challenges, Limitations, Future Directions, and Recommendations

MCTs have been used in clinical nutrition for several decades owing to their distinctive metabolic properties, including rapid intestinal absorption, preferential portal transport, and efficient mitochondrial oxidation. These characteristics underpin their established role in medical nutrition for conditions such as fat malabsorption, ketogenic dietary therapy, and inherited disorders of long-chain fatty-acid oxidation. Despite this long-standing clinical use, broader applications of MCTs in functional nutrition and preventive health strategies remain constrained by regulatory, clinical, and evidentiary limitations, particularly in the context of overweight, obesity, and age-associated malnutrition.

Regulatory approaches to MCT-containing products differ substantially across regions and reflect different interpretations of the available scientific evidence. In Japan, MCTs have been incorporated into functional food systems, including the Foods for Specified Health Uses (FOSHU) framework and the Foods with Function Claims system. Within these paradigms, MCT-containing products may carry functional claims directed at generally healthy adults with elevated body mass index (BMI) and at middle-aged or older populations. Reported functional effects include modest reductions in body fat and waist circumference, increased fat oxidation during daily activities, and improvements in fatigue or physical function when combined with physical activity. These claims are positioned within a health maintenance framework rather than as therapeutic interventions for disease treatment. However, many of the studies supporting these claims rely on relatively short intervention periods and surrogate metabolic endpoints, which limits the ability to infer durable clinical benefits.

In Germany and across the European Union, MCTs are primarily regulated as Foods for Special Medical Purposes (FSMPs). Their use is largely restricted to clearly defined clinical indications, including fat malabsorption syndromes, ketogenic dietary therapies for refractory epilepsy, and inherited disorders of long-chain fatty-acid oxidation. Current European regulatory frameworks do not permit health or function claims for MCTs in the management of overweight, obesity, or age-related sarcopenic malnutrition in otherwise healthy populations. This more restrictive regulatory approach reflects a higher evidentiary threshold for health claims and emphasizes clinically meaningful outcomes rather than short-term metabolic changes.

The United States occupies an intermediate regulatory position. MCTs are widely marketed as conventional food ingredients, dietary supplements, clinical nutrition products, and medical foods. This regulatory flexibility has facilitated broad availability of MCT-containing products, but it has also created a heterogeneous landscape in which products targeting metabolic health, weight management, or cognitive function are frequently marketed despite limited long-term clinical evidence.

In addition to differences in claim substantiation, regulatory evaluation of MCT-containing products must also consider food-safety aspects related to industrial fat processing. Process-related contaminants such as 3-MCPD esters and glycidyl esters may arise during oil-refining and fat-modification processes and are therefore subject to strict regulatory limits, particularly in products intended for vulnerable populations such as infants [129,130,131,132]. These considerations are especially relevant for clinical nutrition products and infant formula, where compositional purity and contaminant control are essential for regulatory approval and safe long-term consumption.

This regulatory divergence must also be considered in the context of population health. In Germany, overweight and obesity remain highly prevalent, with approximately 53.5% of adults classified as overweight (BMI ≥ 25 kg/m^2^) and around 19% meeting criteria for obesity (BMI ≥ 30 kg/m^2^), with prevalence increasing with age and showing pronounced socioeconomic gradients [133]. By contrast, underweight as a population-level indicator of malnutrition is relatively uncommon. Nevertheless, malnutrition remains a clinically relevant and often underdiagnosed condition in hospitalized, chronically ill, and geriatric populations, particularly in the context of acute illness, frailty, and functional decline.

A key limitation underlying both regulatory decisions and clinical recommendations is the quality of the existing evidence base. Many studies supporting functional claims, particularly those conducted within the Japanese regulatory framework, are relatively short-term and rely primarily on surrogate metabolic endpoints such as energy expenditure, fat oxidation rates, or modest changes in body composition. These data are insufficient to establish durable clinical benefits for sustained obesity management or nutritional rehabilitation. Consistent with this assessment, European guideline evaluations—including those of the German Nutrition Society (DGE)—conclude that although partial substitution of long-chain triglycerides with MCTs may induce short-term reductions in body weight or fat mass, current evidence does not demonstrate clinically meaningful long-term effects [72]. Consequently, MCTs cannot presently be recommended as preventive or therapeutic interventions for overweight, obesity, or age-related nutritional decline in the general population.

Addressing these limitations will require a stronger clinical evidence base. Future research should prioritize well-designed, long-term randomized controlled trials in clearly characterized populations, including individuals with elevated BMI, older adults at risk of malnutrition or sarcopenia, and patients with established nutritional deficits. Such studies should employ standardized MCT formulations with clearly defined fatty-acid composition, appropriate comparator fats, and clinically meaningful primary endpoints. Relevant outcomes include sustained changes in body composition, muscle strength, physical performance, frailty status, and quality of life rather than reliance solely on surrogate metabolic markers. Long-term safety and adherence must also be systematically evaluated.

In addition to clinical efficacy, future investigations should integrate health-economic analyses to assess the potential impact of MCT-based interventions on healthcare utilization and costs. Such evaluations may include hospitalization rates, duration of hospital stay, need for nutritional support, long-term care requirements, and other indicators of healthcare resource use. Cost-effectiveness and cost–utility analyses will be essential to determine whether MCT supplementation, if clinically effective, could contribute to reducing healthcare expenditures, particularly in aging populations and in patients at risk of malnutrition-related complications.

Complementary mechanistic studies are also needed to clarify the physiological contexts in which MCT metabolism may confer benefit. Approaches integrating metabolic flux analyses, biomarkers of mitochondrial and peroxisomal fatty-acid oxidation, and interactions with dietary composition, protein intake, and physical activity may help identify patient populations most likely to benefit from MCT supplementation. Ultimately, robust long-term clinical evidence, supported by mechanistic and health-economic data, will be required to determine whether functional claims for MCTs can be justified beyond their established role in specialized medical nutrition and whether they may contribute meaningfully to preventive nutrition and healthy aging strategies.

From a translational perspective, certain elements of the Japanese preventive nutrition model, such as the focus on early nutritional intervention and maintenance of functional capacity in aging populations, may offer conceptual insights for European public health strategies. However, any transfer of such approaches to the EU context would require substantially stronger clinical evidence and alignment with the stricter European standards for substantiation of health claims.

## 7. Conclusions

MCTs possess well-characterized metabolic properties and established clinical indications, including disorders of fat malabsorption, ketogenic dietary therapy, and inherited defects of long-chain fatty-acid β-oxidation. Beyond these indications, MCTs have also been incorporated into broader functional nutrition frameworks, although regulatory approaches differ substantially across regions. In Japan, MCT-containing products are integrated into the Foods for Specified Health Uses (FOSHU) and Foods with Function Claims systems and are positioned within preventive nutrition strategies targeting adults with elevated body mass index and aging populations. In the United States, MCTs are marketed across multiple regulatory categories, including conventional foods, dietary supplements, clinical nutrition formulations, and medical foods. By contrast, in Germany and the European Union, their use remains largely confined to foods for special medical purposes, reflecting a more conservative regulatory framework and a higher evidentiary threshold for health claims.

Despite growing commercial and scientific interest, the current evidence base supporting broader health applications of MCTs remains limited. Many studies rely on short-term interventions and surrogate metabolic endpoints, whereas robust data demonstrating sustained and clinically meaningful benefits for conditions such as overweight, obesity, or age-associated malnutrition are still scarce. Consequently, outside established medical indications, the preventive or therapeutic use of MCTs in the general population cannot currently be recommended on the basis of available evidence.

Future research should therefore prioritize rigorously designed, long-term randomized controlled trials in well-characterized populations. Such studies should employ standardized MCT formulations with clearly defined fatty-acid composition and evaluate clinically relevant outcomes, including body composition, muscle strength, physical performance, frailty status, and quality of life. Complementary mechanistic investigations, health–economic analyses, and structured international evidence synthesis, including data generated within the Japanese and U.S. regulatory contexts, will be essential to determine whether MCTs may contribute meaningfully to preventive nutrition strategies and healthy aging. In this context, insights from the Japanese preventive nutrition model may provide valuable conceptual perspectives. However, any broader adoption within the European regulatory framework will require substantially stronger clinical evidence and alignment with existing standards for substantiation of health claims.

## Figures and Tables

**Table 1:** Nutritional applications of MCTs in gastroenterological and hepatological disorders.

Affected Organ or System [German Guideline Reference]	Functional Impairment	Disorder	Rationale for the Use of MCTs
Liver and gallbladder [47]	Fat malabsorption due to Reduced bile flow into the intestine. Impaired bile acid production.	Cholestatic liver diseases	MCTs do not require emulsification by bile. Their partial water solubility allows direct diffusion into enterocytes and absorption into the portal circulation.
Pancreas [48,49]	Fat malabsorption due to Pancreatic enzyme deficiency (pancreatic lipase).	Exocrine pancreatic insufficiency	MCTs are absorbed without requiring pancreatic lipase.
Small intestine [50]	Fat malabsorption due to intestinal resection.	Short bowel syndrome with significant fat malabsorption	MCTs are partially water-soluble, facilitating rapid intestinal absorption.
Lymphatic system [51]	Defective lymphatic transport.	Chylothorax Chylous ascites Intestinal protein-losing syndrome	MCTs are transported directly via the portal venous system, bypassing lymphatic circulation.

MCTs: medium-chain triglycerides.

**Table 2:** Functional claims of MCFAs.

Target	Effect
Adults with higher BMI	Reduce waist circumference
	Decrease body fat (visceral fat, subcutaneous fat)
	Increase fat burning during daily activities
	Enhance metabolism of ingested lipids
Middle-aged and older adults	Maintain leg muscle strength that declines with age when used in combination with exercise
	Reduce transient physical and mental fatigue experienced in daily life when used in combination with exercise
Adults	Increase fat burning during exercise

**Table 3:** Studies included in the systematic literature reviews.

Functional Claims	Study Design	Subjects	Number of Participants	Test Diet	Control Diet	Exercise Intervention	Duration	Results of Outcomes Related to Functional Claims	Reference
Reduce waist circumference and decrease body fat (visceral and subcutaneous fat)	RCT	Healthy Japanese men and women aged 19 to 58 years	73	Margarine containing 5 g MCT	Margarine containing 5 g blend oils of canola and soybean	-	12 weeks	Waist circumference, body fat percentage, total body fat, subcutaneous fat area, visceral fat area: Significant decrease	[112]
	RCT	Healthy Japanese men and women aged 21 to 59 years	93	Bread containing 14 g MLCT (1.7 g MCFA)	Bread containing 14 g blend oils of canola and soybean	-	12 weeks	Waist circumference, body fat percentage, total body fat, subcutaneous fat area, visceral fat area: Significant decrease	[109]
	RCT	Healthy Japanese men 18 to 20 years	13	Liquid supplement containing 10 g MLCT (10% MCFA)	Liquid supplement containing 10 g soybean oil	-	12 weeks	Body fat percentage: Significant decrease	[113]
	RCT	Healthy Japanese men and women aged 20 to 58 years	78	Bread containing MCT	Bread containing blend oils of canola and soybean	-	12 weeks	Waist circumference, subcutaneous fat areas: Significant decrease (Subgroup analysis for BMI ≥ 23) Visceral fat area: Significant decrease (Subgroup analysis for BMI < 23) Body fat: no significant change	[114]
Increase fat burning during daily activities	RCT	Healthy Japanese men and women aged 36 to 64 years with a high BMI (BMI 25–30)	30	2 g MCT	2 g LCT	Cycle ergometer exercise with a 20-watt load at 50 revolutions per minute for 30 min	2 weeks	Fat oxidation rate: Significant increase Respiratory exchange ratio: Significant decrease	[92]
	RCT	Healthy Japanese men and women aged 35 to 64 years with a high BMI (BMI 25–30)	30	14 g MLCT (1.6 g MCFA)	14 g rapeseed oil	Cycle ergometer exercise with a 20-watt load at 50 revolutions per minute for 30 min	4 weeks	Fat oxidation rate: Significant increase Respiratory exchange ratio: Trend toward significant decrease	[115]
Enhance metabolism of ingested lipids	RCT	Healthy Japanese men and women aged 50.5 ± 8.0 years with a high BMI (BMI 25–30) (mean ± SD)	30	14 g MLCT (1.6 g MCFA)	14 g rapeseed oil	-	4 weeks	Metabolic rate of ingested LCTs: Significant increase	[116]
	RCT	Healthy Japanese men and women aged 35 to 64 years with a high BMI (BMI 25–30)	30	2 g MCT	2 g rapeseed oil	-	2 weeks	Metabolic rate of ingested LCTs: Significant increase	[117]
Maintain leg muscle strength that declines with age when used in combination with exercise	RCT	Healthy Japanese men and women aged 60 to 75 years	120	① 6 g MCT (formulation rich in decanoic acid) ② 2 g MCT (formulation rich in octanoic acid), 4g rapeseed oil ③ 6 g MCT (formulation rich in octanoic acid)	6 g rapeseed oil	Walking exercise was incorporated twice a week for 40 ± 10 min per session	12 weeks	Right knee extensor strength: Significant improvements in groups ①, ②, and ③ Left knee extensor strength: Significant improvements in groups ①, ②, and ③ Grip strength: No significant differences	[85]
Reduce transient physical and mental fatigue experienced in daily life when used in combination with exercise	RCT	Healthy Japanese men and women aged 60 to 74 years	120	① 6 g MCT (formulation rich in decanoic acid) ② 2 g MCT (formulation rich in octanoic acid), 4 g rapeseed oil ③ 6 g MCT (formulation rich in octanoic acid)	6 g rapeseed oil	Walking exercise was incorporated twice a week for 40 ± 10 min per session	12 weeks	Physical fatigue (SF-36 Vitality score): Significant improvements in groups ①, ②, and ③ Mental fatigue (SF-36 Mental Health score): Significant improvements in groups ① and ② Mental fatigue (SF-36 Mental Component Summary score): Significant improvements in groups ①, ②, and ③	[118]
Increase fat burning during exercise	RCT	Healthy Japanese men and women aged 35 to 64 years with a high BMI (BMI 25–30)	30	2 g MCT	2 g LCT	Cycle ergometer exercise at a workload of 20 W	2 weeks	Fat oxidation rate: Significant increase Maximal fat oxidation rate: Trend toward increase Respiratory exchange ratio: Significant decrease	[115]
	RCT	Healthy Japanese men and women aged 40 to 60 years with an average BMI of 22.8 ± 1.6 (mean ± SD)	30	Beverage containing 6 g of MCT rich in decanoic acid (DAR) Beverage containing 6 g of MCT rich in octanoic acid (OAR)	Beverage containing no fat.	Cycle ergometer exercise starting with 3 min at 20 W (Fixed load), followed by incremental workload increases of 13 W/min for males and 10 W/min for females	2 weeks	Fat oxidation amount: Significantly increased with OAR during fixed load Maximal fat oxidation rate: Significantly increased with OAR during fixed load Respiratory exchange ratio: Significantly decreased with OAR during fixed load	[91]
	RCT	Healthy Japanese women aged 20 to 24 years with an average BMI of 22.7 ± 2.1 (mean ± SD)	8	Jelly drink containing 6 g of MCT	An isoenergetic placebo jelly drink containing carbohydrates without fat	Cycle ergometer exercise with a 5 min warm-up at 40 W, then cycling at 50–60 rpm and 50% peak VO_2_ for 40 min, followed by workload increase to 70% peak VO_2_ until exhaustion	2 weeks	Fat oxidation amount: Significantly increased at 50% peak VO_2_ Fat oxidation rate: Significantly increased at 70% peak VO_2_ Respiratory exchange ratio: Significantly decreased at 50% peak VO_2_	[90]
	RCT	Healthy Japanese men and women aged 21 to 28 years with an average BMI of 22.2 ± 2.3 (mean ± SD)	8	Baked meal containing 6 g of MCT	Baked meal containing 6 g of LCT	Cycle ergometer exercise with a 5 min warm-up at 40 W, then cycling at 50–60 rpm and 50% peak VO_2_ for 40 min, followed by workload increase to 80% peak VO_2_ until exhaustion	2 weeks	Fat oxidation rate: Trend toward increased values Respiratory exchange ratio: No significant difference	[89]
	RCT	Japanese men and women aged 35–64 years, with a high BMI (BMI 25–30)	30	14 g MLCT (1.6 g MCFA)	14 g rapeseed oil	Cycle ergometer exercise with a 20-watt load at 50 revolutions per minute for 30 min.	4 weeks	Fat oxidation rate: Significantly increased Respiratory exchange ratio: Trend toward decreased values	[115]

The outcomes related to functional claims were selected based on the descriptions in the systematic literature review.

**Table 4:** Comparative Overview of Regulatory Frameworks for MCTs in Germany and Japan.

Category	Germany	Japan
**Regulatory framework**	EU Regulation No 609/2013 and Delegated Regulation 2016/128; complemented by Regulation on Food for Specific Groups (Lebensmittel für bestimmte Verbrauchergruppen-Verordnung, LMBVV).	Foods in General vs. Foods with Health Claims which is subdivided into Foods with Nutrient Function Claims, FOSHU, Foods with Function Claims.
**Regulatory classification**	FSMPs: nutritionally incomplete foods with a nutrient-adapted formulation specific for disease or medical condition; not suitable as the sole source of nutrition.	FOSHU or Foods with Function Claims, also marketed as Foods in General.
**Target group/population**	Patients with diagnosed medical conditions (e.g., short bowel syndrome, pancreatic insufficiency, neurological or metabolic disorders) and to be used under medical supervision.	Healthy individuals or at-risk populations of lifestyle-related diseases.
**Approval process**	Manufacturers submit notifications of FSMPs to the BVL in accordance with Section 3 of the Regulation on Food for Specific Groups (LMBVV). After formal verification, notifications are forwarded to the relevant state authorities, which conduct market surveillance and sample-based inspections.	FOSHU: national review for safety and efficacy, with approval from the Consumer Affairs Agency; Foods with Function Claims: manufacturer submits scientific evidence before marketing.
**Evidence**	EFSA guidance (Article 3 of Regulation (EU) No 609/2013) specifies that manufacturers must, upon request, provide a structured dossier covering product characterization, intended use, target patients, disease context, dietary management role, and conditions for medical supervision.	FOSHU: national efficacy review; Foods with Function Claims: scientific evidence (e.g., systematic literature reviews) submitted by manufacturer.
**Regulatory support**	EFSA-guided dossier requirements and the BVL/BfArM position paper provide a structured framework and decision tree to support consistent classification of products as FSMPs, offering orientation for manufacturers and state authorities and promoting uniform nationwide regulatory practice.	The Consumer Affairs Agency provides guidance and review procedures for FOSHU and Foods with Function Claims, establishing a systematic, evidence-based framework that supports consistent product classification, offers orientation for manufacturers, and promotes reliable nationwide regulatory practice for functional foods.

FSMPs: Food for Special Medical Purposes; FOSHU: Food for Specified Health Uses; EFSA: European Food Safety Authority; BVL: The Federal Office of Consumer Protection and Food Safety; BfArM: The Federal Institute for Drugs and Medical Devices; LMBVV: Food for Specific Groups.

## Data Availability

No new data were created or analyzed in this study.

## References

[1] Schönfeld P., Wojtczak L. (2016). Short- and medium-chain fatty acids in energy metabolism: The cellular perspective. J. Lipid Res..

[2] Rinaldo P., Welch R.D., Previs S.F., Schmidt-Sommerfeld E., Gargus J.J., O’Shea J.J., Zinn A.B. (1991). Ethylmalonic/adipic aciduria: Effects of oral medium-chain triglycerides, carnitine, and glycine on urinary excretion of organic acids, acylcarnitines, and acylglycines. Pediatr. Res..

[3] Tserng K.Y., Griffin R.L., Kerr D.S. (1996). Distinction of dicarboxylic aciduria due to medium-chain triglyceride feeding from that due to abnormal fatty acid oxidation and fasting in children. Metabolism.

[4] Sailer D., Berg G. (1974). Medium-chain triglycerides. Clinical physiology and application. Z. Ernahrungswiss..

[5] Heidt C., Newport M., Kämmerer U. (2025). Mittelkettige Triglyceride und deren Einsatz in der Ernährungsmedizin. Aktuelle Ernährungsmedizin.

[6] Augustin K., Khabbush A., Williams S., Eaton S., Orford M., Cross J.H., Heales S.J.R., Walker M.C., Williams R.S.B. (2018). Mechanisms of action for the medium-chain triglyceride ketogenic diet in neurological and metabolic disorders. Lancet Neurol..

[7] Jeukendrup A.E., Thielen J.J., Wagenmakers A.J., Brouns F., Saris W.H. (1998). Effect of medium-chain triacylglycerol and carbohydrate ingestion during exercise on substrate utilization and subsequent cycling performance. Am. J. Clin. Nutr..

[8] Greenberger N.J., Skillman T.G. (1969). Medium-chain triglycerides. N. Engl. J. Med..

[9] Edem D.O. (2002). Palm oil: Biochemical, physiological, nutritional, hematological, and toxicological aspects: A review. Plant Foods Hum. Nutr..

[10] Babayan V.K. (1968). Medium-chain triglycerides--their composition, preparation, and application. J. Am. Oil Chem. Soc..

[11] Watanabe S., Tsujino S. (2022). Applications of Medium-Chain Triglycerides in Foods. Front. Nutr..

[12] Duranova H., Kuzelova L., Fialkova V., Simora V., Kovacikova E., Joanidis P., Borotova P., Straka D., Hoskin R.T., Moncada M. (2025). Coconut-sourced MCT oil: Its potential health benefits beyond traditional coconut oil. Phytochem. Rev..

[13] Liao T.H., Hamosh P., Hamosh M. (1984). Fat digestion by lingual lipase: Mechanism of lipolysis in the stomach and upper small intestine. Pediatr. Res..

[14] DeNigris S.J., Hamosh M., Kasbekar D.K., Fink C.S., Lee T.C., Hamosh P. (1985). Secretion of human gastric lipase from dispersed gastric glands. Biochim. Biophys. Acta.

[15] Tantibhedhyangkul P., Hashim S.A. (1975). Medium-chain triglyceride feeding in premature infants: Effects on fat and nitrogen absorption. Pediatrics.

[16] Roy C.C., Ste-Marie M., Chartrand L., Weber A., Bard H., Doray B. (1975). Correction of the malabsorption of the preterm infant with a medium-chain triglyceride formula. J. Pediatr..

[17] Cohen M.I., Gartner L.M. (1971). The use of medium-chain triglycerides in the management of biliary atresia. J. Pediatr..

[18] Hopman W.P., Jansen J.B., Rosenbusch G., Lamers C.B. (1984). Effect of equimolar amounts of long-chain triglycerides and medium-chain triglycerides on plasma cholecystokinin and gallbladder contraction. Am. J. Clin. Nutr..

[19] Houten S.M., Wanders R.J. (2010). A general introduction to the biochemistry of mitochondrial fatty acid β-oxidation. J. Inherit. Metab. Dis..

[20] Pereyra A.S., McLaughlin K.L., Buddo K.A., Ellis J.M. (2023). Medium-chain fatty acid oxidation is independent of l-carnitine in liver and kidney but not in heart and skeletal muscle. Am. J. Physiol. Gastrointest. Liver Physiol..

[21] Sidossis L.S., Stuart C.A., Shulman G.I., Lopaschuk G.D., Wolfe R.R. (1996). Glucose plus insulin regulate fat oxidation by controlling the rate of fatty acid entry into the mitochondria. J. Clin. Investig..

[22] McGarry J.D., Meier J.M., Foster D.W. (1973). The effects of starvation and refeeding on carbohydrate and lipid metabolism in vivo and in the perfused rat liver. The relationship between fatty acid oxidation and esterification in the regulation of ketogenesis. J. Biol. Chem..

[23] Foster D.W. (2012). Malonyl-CoA: The regulator of fatty acid synthesis and oxidation. J. Clin. Investig..

[24] Papamandjaris A.A., MacDougall D.E., Jones P.J. (1998). Medium chain fatty acid metabolism and energy expenditure: Obesity treatment implications. Life Sci..

[25] Kanta J.M., Lundsgaard A.M., Havelund J.F., Armour S.L., Bæk O., Nguyen D.N., Richter E.A., Knudsen J.G., Kleinert M., Færgeman N.J. (2025). Metabolic effects of medium-chain triacylglycerol consumption are preserved in obesity. Am. J. Physiol. Endocrinol. Metab..

[26] Lin T.Y., Liu H.W., Hung T.M. (2021). The Ketogenic Effect of Medium-Chain Triacylglycerides. Front. Nutr..

[27] Ranea-Robles P., Houten S.M. (2023). The biochemistry and physiology of long-chain dicarboxylic acid metabolism. Biochem. J..

[28] Semba R.D., Trehan I., Li X., Moaddel R., Ordiz M.I., Maleta K.M., Kraemer K., Shardell M., Ferrucci L., Manary M. (2017). Environmental Enteric Dysfunction is Associated with Carnitine Deficiency and Altered Fatty Acid Oxidation. EBioMedicine.

[29] Villarreal-Pérez J.Z., Villarreal-Martínez J.Z., Lavalle-González F.J., Torres-Sepúlveda Mdel R., Ruiz-Herrera C., Cerda-Flores R.M., Castillo-García E.R., Rodríguez-Sánchez I.P., Martínez de Villarreal L.E. (2014). Plasma and urine metabolic profiles are reflective of altered beta-oxidation in non-diabetic obese subjects and patients with type 2 diabetes mellitus. Diabetol. Metab. Syndr..

[30] Gregersen N., Ingerslev J. (1979). The excretion of C6-C10-dicarboxylic acids in the urine of newborn infants during starvation. Evidence for omega-oxidation of fatty acids in the newborn. Acta Paediatr. Scand..

[31] Lee R.B., Duncan L.L., Roth K.S. (2001). Dietary medium-chain triglycerides: A source of urinary dicarboxylic acids and diagnostic confusion. Clin. Pediatr..

[32] Wada F., Usami M. (1977). Studies on fatty acid omega-oxidation. Antiketogenic effect and gluconeogenicity of dicarboxylic acids. Biochim. Biophys. Acta.

[33] Jadhav H.B., Annapure U.S. (2023). Triglycerides of medium-chain fatty acids: A concise review. J. Food Sci. Technol..

[34] Ho Ahn J., Hwan Jung K., Seok Lim E., Min Kim S., Ok Han S., Um Y. (2023). Recent advances in microbial production of medium chain fatty acid from renewable carbon resources: A comprehensive review. Bioresour. Technol..

[35] Nimbkar S., Leena M.M., Moses J.A., Anandharamakrishnan C. (2022). Medium chain triglycerides (MCT): State-of-the-art on chemistry, synthesis, health benefits and applications in food industry. Compr. Rev. Food Sci. Food Saf..

[36] Boulos Z., Duceppe J.-S., Penney C. (2016). Method for the Preparation of Triglycerides of Medium-Chain Length Fatty Acids. U.S. Patent.

[37] Tojo T., Sugihara S. (2017). Method of Producing Medium Chain Fatty Acid Using Beta-Ketoacyl-ACP Synthase. U.S. Patent.

[38] Langone M.A., Sant’Anna G.L. (1999). Enzymatic synthesis of medium-chain triglycerides in a solvent-free system. Appl. Biochem. Biotechnol..

[39] Salvador S.D., Apostol G.C. (2019). Preparation and Composition of Medium Chain Triglycerides Containing Substantial Amount of Lauric Acid. U.S. Patent.

[40] Galante J.H., Clauss S.L., Bernhardt R.J., Schultz A.K. (2010). Process for Enzymatic Production of Triglycerides. U.S. Patent.

[41] Schoerken U., Meyer C., Horlacher P., Both S. (2007). Process for the Enzymatic Synthesis of Triglycerides. U.S. Patent.

[42] Dragan V. (2021). Process for Enzymatic Production of Triglycerides. U.S. Patent.

[43] Rongione J.C., Heydinger-Galante J. (2015). Elimination of Organohalo and Oxirane Species in Carboxylic Acid Ester Streams. U.S. Patent.

[44] Ozaki T., Takimura Y. (2015). Method for Producing Lipid. U.S. Patent.

[45] Lochmann D., Reyer S., Stehr M. (2024). Process for Producing High-Grade Fatty Acide Polyol Esters, Particularly Fatty Acid Glycerol Esters. U.S. Patent.

[46] Lochmann D., Reyer S., Stehr M. (2024). Process for Producing High-Grade Tricaprylin. U.S. Patent.

[47] Grothues D., Engelhardt H., Genzel-Boroviczeny O., Gnädig M., Harm M., Hörning A., Muensterer O., Pfister E.-D., Rodeck B., Sokollik C. (2020). S2k Leitlinie Cholestase im Neugeborenenalter, AWMF-Register Nr. 068/015. AWMF-Register.

[48] Beyer G., Hoffmeister A., Michl P., Gress T.M., Huber W., Algül H., Neesse A., Meining A., Seufferlein T.W., Rosendahl J. (2022). S3-leitlinie pankreatitis–leitlinie der deutschen gesellschaft für gastroenterologie, verdauungs-und stoffwechselkrankheiten (DGVS)–september 2021–AWMF registernummer 021-003. Z. Gastroenterol..

[49] Hammermann J., Claßen M., Schmidt S., Bend J., Ballmann M., Baumann I., Bremer W., Ellemunter H., Felbor U., Hahn G. (2020). S3-Leitlinie: Mukoviszidose bei Kindern in den Ersten Beiden Lebensjahren, Diagnostik und Therapie. https://register.awmf.org/de/leitlinien/detail/026-024.

[50] Lamprecht G., Pape U.-F., Witte M., Pascher A., DGEM Steering Committee (2014). S3-Leitlinie der Deutschen Gesellschaft für Ernährungsmedizin e. V. in Zusammenarbeit mit der AKE, der GESKES und der DGVS. Aktuelle Ernährungsmedizin.

[51] Lymphologen G.D. (2017). S2k Leitlinie-Diagnostik und Therapie der Lymphödeme.

[52] Cahill G.F. (2006). Fuel metabolism in starvation. Annu. Rev. Nutr..

[53] Kossoff E.H., Zupec-Kania B.A., Auvin S., Ballaban-Gil K.R., Christina Bergqvist A.G., Blackford R., Buchhalter J.R., Caraballo R.H., Cross J.H., Dahlin M.G. (2018). Optimal clinical management of children receiving dietary therapies for epilepsy: Updated recommendations of the International Ketogenic Diet Study Group. Epilepsia Open.

[54] Huttenlocher P.R., Wilbourn A.J., Signore J.M. (1971). Medium-chain triglycerides as a therapy for intractable childhood epilepsy. Neurology.

[55] Huttenlocher P.R. (1976). Ketonemia and seizures: Metabolic and anticonvulsant effects of two ketogenic diets in childhood epilepsy. Pediatr. Res..

[56] Liu Y.M., Wang H.S. (2013). Medium-chain triglyceride ketogenic diet, an effective treatment for drug-resistant epilepsy and a comparison with other ketogenic diets. Biomed. J..

[57] Neal E.G., Chaffe H., Schwartz R.H., Lawson M.S., Edwards N., Fitzsimmons G., Whitney A., Cross J.H. (2009). A randomized trial of classical and medium-chain triglyceride ketogenic diets in the treatment of childhood epilepsy. Epilepsia.

[58] Boison D. (2017). New insights into the mechanisms of the ketogenic diet. Curr. Opin. Neurol..

[59] Schoeler N.E., Cross J.H., Sander J.W., Sisodiya S.M. (2013). Can we predict a favourable response to Ketogenic Diet Therapies for drug-resistant epilepsy?. Epilepsy Res..

[60] Hughes S.D., Kanabus M., Anderson G., Hargreaves I.P., Rutherford T., O’Donnell M., Cross J.H., Rahman S., Eaton S., Heales S.J. (2014). The ketogenic diet component decanoic acid increases mitochondrial citrate synthase and complex I activity in neuronal cells. J. Neurochem..

[61] Wlaź P., Socała K., Nieoczym D., Żarnowski T., Żarnowska I., Czuczwar S.J., Gasior M. (2015). Acute anticonvulsant effects of capric acid in seizure tests in mice. Prog. Neuropsychopharmacol. Biol. Psychiatry.

[62] Chang P., Augustin K., Boddum K., Williams S., Sun M., Terschak J.A., Hardege J.D., Chen P.E., Walker M.C., Williams R.S. (2016). Seizure control by decanoic acid through direct AMPA receptor inhibition. Brain.

[63] Khabbush A., Orford M., Tsai Y.C., Rutherford T., O’Donnell M., Eaton S., Heales S.J.R. (2017). Neuronal decanoic acid oxidation is markedly lower than that of octanoic acid: A mechanistic insight into the medium-chain triglyceride ketogenic diet. Epilepsia.

[64] Wright S.K., Wilson M.A., Walsh R., Lo W.B., Mundil N., Agrawal S., Philip S., Seri S., Greenhill S.D., Woodhall G.L. (2020). Abolishing spontaneous epileptiform activity in human brain tissue through AMPA receptor inhibition. Ann. Clin. Transl. Neurol..

[65] Yelshanskaya M.V., Singh A.K., Narangoda C., Williams R.S.B., Kurnikova M.G., Sobolevsky A.I. (2022). Structural basis of AMPA receptor inhibition by trans-4-butylcyclohexane carboxylic acid. Br. J. Pharmacol..

[66] Vockley J. (2020). Long-chain fatty acid oxidation disorders and current management strategies. Am. J. Manag. Care.

[67] Spiekerkoetter U., Lindner M., Santer R., Grotzke M., Baumgartner M.R., Boehles H., Das A., Haase C., Hennermann J.B., Karall D. (2009). Treatment recommendations in long-chain fatty acid oxidation defects: Consensus from a workshop. J. Inherit. Metab. Dis..

[68] Van Calcar S.C., Sowa M., Rohr F., Beazer J., Setlock T., Weihe T.U., Pendyal S., Wallace L.S., Hansen J.G., Stembridge A. (2020). Nutrition management guideline for very-long chain acyl-CoA dehydrogenase deficiency (VLCAD): An evidence- and consensus-based approach. Mol. Genet. Metab..

[69] Behrend A.M., Harding C.O., Shoemaker J.D., Matern D., Sahn D.J., Elliot D.L., Gillingham M.B. (2012). Substrate oxidation and cardiac performance during exercise in disorders of long chain fatty acid oxidation. Mol. Genet. Metab..

[70] Gillingham M.B., Scott B., Elliott D., Harding C.O. (2006). Metabolic control during exercise with and without medium-chain triglycerides (MCT) in children with long-chain 3-hydroxy acyl-CoA dehydrogenase (LCHAD) or trifunctional protein (TFP) deficiency. Mol. Genet. Metab..

[71] Peña-Quintana L., Correcher-Medina P. (2024). Nutritional Management of Patients with Fatty Acid Oxidation Disorders. Nutrients.

[72] Leitlinie E. (2015). Fettzufuhr und Prävention Ausgewählter Ernährungsmitbedingter Krankheiten. https://www.dge.de/fileadmin/dok/presse/meldungen/2011-2018/Gesamt-DGE-Leitlinie-Fett-2015.pdf.

[73] Schek A., Braun H., Carlsohn A., Großhauser M., König D., Lampen A., Mosler S., Nieß A., Oberritter H., Schäbethal K. (2019). Fette in der Sporternährung-Position der Arbeitsgruppe Sporternährung der Deutschen Gesellschaft für Ernährung e. V. (DGE). https://www.dge.de/fileadmin/dok/gesunde-ernaehrung/gezielte-ernaehrung/sportler-innen/EU09_2019_M538-M545.pdf.

[74] Jeukendrup A.E., Aldred S. (2004). Fat supplementation, health, and endurance performance. Nutrition.

[75] Clegg M.E. (2010). Medium-chain triglycerides are advantageous in promoting weight loss although not beneficial to exercise performance. Int. J. Food Sci. Nutr..

[76] Xue Q.L., Bandeen-Roche K., Varadhan R., Zhou J., Fried L.P. (2008). Initial manifestations of frailty criteria and the development of frailty phenotype in the Women’s Health and Aging Study II. J. Gerontol. A Biol. Sci. Med. Sci..

[77] Higashiguchi T., Arai H., Claytor L.H., Kuzuya M., Kotani J., Lee S.D., Michel J.P., Nogami T., Peng N. (2017). Taking action against malnutrition in Asian healthcare settings: An initiative of a Northeast Asia Study Group. Asia Pac. J. Clin. Nutr..

[78] Nosaka N., Adachi K., Kawashima Y., Suzuki H., Hayashi S., Aoyama T., Nakamura T. (2010). Effect of ingestion of medium-chain fatty acids on serum albumin in the elderly with protein-energy malnutrition (PEM). J. Jpn. Soc. Clin. Nutr..

[79] Sekine S., Terada S., Aoyama T. (2013). Medium-chain triacylglycerol suppresses the decrease of plasma albumin level through the insulin-Akt-mTOR pathway in the livers of malnourished rats. J. Nutr. Sci. Vitaminol..

[80] Nonaka H., Ohue-Kitano R., Masujima Y., Igarashi M., Kimura I. (2022). Dietary Medium-Chain Triglyceride Decanoate Affects Glucose Homeostasis Through GPR84-Mediated GLP-1 Secretion in Mice. Front. Nutr..

[81] Evans D.C., Corkins M.R., Malone A., Miller S., Mogensen K.M., Guenter P., Jensen G.L. (2021). The Use of Visceral Proteins as Nutrition Markers: An ASPEN Position Paper. Nutr. Clin. Pract..

[82] Cederholm T., Jensen G.L., Correia M., Gonzalez M.C., Fukushima R., Higashiguchi T., Baptista G., Barazzoni R., Blaauw R., Coats A. (2019). GLIM criteria for the diagnosis of malnutrition—A consensus report from the global clinical nutrition community. Clin. Nutr..

[83] Jensen G.L., Cederholm T., Correia M., Gonzalez M.C., Fukushima R., Higashiguchi T., de Baptista G.A., Barazzoni R., Blaauw R., Coats A.J.S. (2019). GLIM Criteria for the Diagnosis of Malnutrition: A Consensus Report From the Global Clinical Nutrition Community. JPEN J. Parenter. Enter. Nutr..

[84] Ezaki O., Abe S. (2023). Medium-chain triglycerides (8:0 and 10:0) increase muscle mass and function in frail older adults: A combined data analysis of clinical trials. Front. Nutr..

[85] Kojima K., Ishikawa H., Watanabe S., Nosaka N., Mutoh T. (2023). A Randomized, Double-Blind, Controlled Trial Assessing If Medium-Chain Triglycerides in Combination with Moderate-Intensity Exercise Increase Muscle Strength in Healthy Middle-Aged and Older Adults. Nutrients.

[86] Yoshimura Y., Nagano F., Matsumoto A., Shimazu S., Shiraishi A., Kido Y., Bise T., Hamada T., Yoneda K. (2025). Synergistic Effects of Medium-Chain Triglyceride Supplementation and Resistance Training on Physical Function and Muscle Health in Post-Stroke Patients. Nutrients.

[87] Décombaz J., Arnaud M.J., Milon H., Moesch H., Philippossian G., Thélin A.L., Howald H. (1983). Energy metabolism of medium-chain triglycerides versus carbohydrates during exercise. Eur. J. Appl. Physiol. Occup. Physiol..

[88] Massicotte D., Péronnet F., Brisson G.R., Hillaire-Marcel C. (1992). Oxidation of exogenous medium-chain free fatty acids during prolonged exercise: Comparison with glucose. J. Appl. Physiol..

[89] Nosaka N., Suzuki Y., Nagatoishi A., Kasai M., Wu J., Taguchi M. (2009). Effect of ingestion of medium-chain triacylglycerols on moderate- and high-intensity exercise in recreational athletes. J. Nutr. Sci. Vitaminol..

[90] Nosaka N., Suzuki Y., Suemitsu H., Kasai M., Kato K., Taguchi M. (2018). Medium-chain Triglycerides with Maltodextrin Increase Fat Oxidation during Moderate-intensity Exercise and Extend the Duration of Subsequent High-intensity Exercise. J. Oleo Sci..

[91] Nosaka N., Tsujino S., Honda K., Suemitsu H., Kato K. (2020). Enhancement of Fat Oxidation during Submaximal Exercise in Sedentary Persons: Variations by Medium-Chain Fatty Acid Composition and Age Group. Lipids.

[92] Tsujino S., Nosaka N., Sadamitsu S., Kato K. (2022). Effect of Continuous Ingestion of 2 g of Medium-Chain Triglycerides on Substrate Metabolism during Low-Intensity Physical Activity. Nutrients.

[93] Fukazawa A., Koike A., Karasawa T., Tsutsui M., Kondo S., Terada S. (2020). Effects of a Ketogenic Diet Containing Medium-Chain Triglycerides and Endurance Training on Metabolic Enzyme Adaptations in Rat Skeletal Muscle. Nutrients.

[94] University of Pennsylvania Press (1968). Medium Chain Triglycerides.

[95] Eckart J., Adolph M., van der Mühlen U., Naab V. (1980). Fat emulsions containing medium chain triglycerides in parenteral nutrition of intensive care patients. JPEN J. Parenter. Enter. Nutr..

[96] Crowe P.J., Dennison A.R., Royle G.T. (1985). A new intravenous emulsion containing medium-chain triglyceride: Studies of its metabolic effects in the perioperative period compared with a conventional long-chain triglyceride emulsion. JPEN J. Parenter. Enter. Nutr..

[97] Tantibhedhyangkul P., Hashim S.A. (1971). Clinical and physiologic aspects of medium-chain triglycerides: Allviation of steatorrhea in premature infants. Bull. N. Y. Acad. Med..

[98] Jensen C., Buist N.R., Wilson T. (1986). Absorption of individual fatty acids from long chain or medium chain triglycerides in very small infants. Am. J. Clin. Nutr..

[99] Hamosh M., Bitman J., Wood L., Hamosh P., Mehta N.R. (1985). Lipids in milk and the first steps in their digestion. Pediatrics.

[100] Jensen R.G. (1999). Lipids in human milk. Lipids.

[101] Fortier M., Castellano C.A., Croteau E., Langlois F., Bocti C., St-Pierre V., Vandenberghe C., Bernier M., Roy M., Descoteaux M. (2019). A ketogenic drink improves brain energy and some measures of cognition in mild cognitive impairment. Alzheimers Dement..

[102] Taylor M.K., Sullivan D.K., Mahnken J.D., Burns J.M., Swerdlow R.H. (2018). Feasibility and efficacy data from a ketogenic diet intervention in Alzheimer’s disease. Alzheimers Dement..

[103] Berg G., Ohnhaus E., Leis D. (1968). Zur Behandlung von Resorptionsstörungen mit mittelkettigen Triglyceriden (MCT) als Bestandteil einer Formuladiät. Med. Ernähr..

[104] EFSA Panel on Dietetic Products, Nutrition and Allergies (NDA) (2021). Scientific and technical guidance on foods for special medical purposes in the context of Article 3 of Regulation (EU) No 609/2013. EFSA J..

[105] Bundesamt für Verbraucherschutz und Lebensmittelsicherheit Positionspapier des BVL und des BfArM. https://www.bfarm.de/SharedDocs/Downloads/DE/Arzneimittel/Zulassung/ZulRelThemen/abgrenzung/Positionspapier-BVL-BfArM-2016.pdf?__blob=publicationFile.

[106] Consumer Affairs Agency What Is the Labeling System for Nutrition and Health Claims?. https://www.caa.go.jp/policies/policy/food_labeling/health_and_nutrition_labelling.

[107] Consumer Affairs Agency About Foods with Nutrient Function Claims. https://www.caa.go.jp/policies/policy/food_labeling/foods_with_nutrient_function_claims.

[108] Consumer Affairs Agency About Foods for Specified Health Uses. https://www.caa.go.jp/policies/policy/food_labeling/foods_for_specified_health_uses.

[109] Kasai M., Nosaka N., Maki H., Negishi S., Aoyama T., Nakamura M., Suzuki Y., Tsuji H., Uto H., Okazaki M. (2003). Effect of dietary medium- and long-chain triacylglycerols (MLCT) on accumulation of body fat in healthy humans. Asia Pac. J. Clin. Nutr..

[110] Consumer Affairs Agency What Are “Foods with Function Claims”. https://www.caa.go.jp/policies/policy/food_labeling/information/pamphlets/pdf/151224_1.pdf.

[111] Consumer Affairs Agency Search for Notified Information on Foods with Functionally Claimed Properties. https://www.caa.go.jp/policies/policy/food_labeling/foods_with_function_claims/search.

[112] Nosaka N., Maki H., Suzuki Y., Haruna H., Ohara A., Kasai M., Tsuji H., Aoyama T., Okazaki M., Igarashi O. (2003). Effects of margarine containing medium-chain triacylglycerols on body fat reduction in humans. J. Atheroscler. Thromb..

[113] Matsuo T., Matsuo M., Kasai M., Takeuchi H. (2001). Effects of a liquid diet supplement containing structured medium- and long-chain triacylglycerols on bodyfat accumulation in healthy young subjects. Asia Pac. J. Clin. Nutr..

[114] Tsuji H., Kasai M., Takeuchi H., Nakamura M., Okazaki M., Kondo K. (2001). Dietary medium-chain triacylglycerols suppress accumulation of body fat in a double-blind, controlled trial in healthy men and women. J. Nutr..

[115] Tsujino S., Nosaka N., Ando N., Sadamitsu S., Kato K. (2023). Continuous ingestion of medium- and long-chain triglycerides enhances fat oxidation during physical activity in subjects with a body mass index from 25 to less than 30: A randomized, placebo-controlled, double-blind crossover study. Jpn. Pharmacol. Ther..

[116] Nosaka N., Tsujino S., Sadamitsu S., Ando N., Kato K. (2023). Ingestion of triglycerides containing medium- and long-chain fatty acids can increase metabolism of ingested long-chain triglycerides in overweight persons. Front. Nutr..

[117] Nosaka N., Tsujino S., Kato K. (2022). Short-Term Ingestion of Medium-Chain Triglycerides Could Enhance Postprandial Consumption of Ingested Fat in Individuals with a Body Mass Index from 25 to Less than 30: A Randomized, Placebo-Controlled, Double-Blind Crossover Study. Nutrients.

[118] Ishikawa H., Kojima K., Watanabe S., Nosaka N., Mutoh T. (2023). Effect of medium-chain triglycerides supplements and walking on health-related quality of life in sedentary, healthy middle-aged, and older adults with low BMIs: A randomized, double-blind, placebo-controlled, parallel-group trial. Front. Nutr..

[119] United States Government Food Additives Amendment of 1958. https://www.congress.gov/bill/85th-congress/house-bill/13254/text.

[120] U.S. Food and Drug Administration (1958). Substances Generally Recognized as Safe. Fed. Regist..

[121] U.S. Food and Drug Administration (1997). Substances Generally Recognized as Safe (GRAS); Proposed Rule. Fed. Regist..

[122] Food and Drug Administration GRAS Notice No. GRN 449: Medium-Chain Triglycerides. https://hfpappexternal.fda.gov/scripts/fdcc/index.cfm?set=grasnotices&id=449.

[123] Food and Drug Administration GRAS Notice No. GRN 1049: Medium-Chain Triglycerides for Use in Infant Formula. https://www.fda.gov/media/162293/download.

[124] United States C. Dietary Supplement Health and Education Act of 1994. https://ods.od.nih.gov/About/DSHEA_Wording.aspx.

[125] Food and Drug Administration Current Good Manufacturing Practice in Manufacturing, Packaging, Labeling, or Holding Operations for Dietary Supplements. https://www.ecfr.gov/current/title-21/chapter-I/subchapter-B/part-111.

[126] Food and Drug Administration Food Labeling Regulations. https://www.ecfr.gov/current/title-21/chapter-I/subchapter-B/part-101?toc=1.

[127] U.S. Congress Definition of Medical Foods.

[128] Food and Drug Administration (2016). Guidance for Industry: Frequently Asked Questions About Medical Foods. https://www.fda.gov/regulatory-information/search-fda-guidance-documents/guidance-industry-frequently-asked-questions-about-medical-foods-third-edition.

[129] European Commission (2018). Commission Regulation (EU) 2018/290 of 26 February 2018 amending Regulation (EC) No 1881/2006 as regards maximum levels of glycidyl fatty acid esters in vegetable oils and fats, infant formula, follow-on formula and foods for special medical purposes intended for infants and young children. Off. J. Eur. Union.

[130] European Food Safety Authority (2016). Risks for Human Health Related to the Presence of 3- and 2-Monochloropropanediol (MCPD), and Their Fatty Acid Esters, and Glycidyl Fatty Acid Esters in Food.

[131] European Commission (2023). Commission Regulation (EU) 2023/915 on maximum levels for certain contaminants in food and repealing Regulation (EC) No 1881/2006. Off. J. Eur. Union.

[132] European Commission (2020). Commission Regulation (EU) 2020/1322 amending Regulation (EC) No 1881/2006 as regards maximum levels of 3-MCPD, 3-MCPD fatty acid esters and glycidyl fatty acid esters in certain foods. Off. J. Eur. Union.

[133] Schienkiewitz A., Kuhnert R., Blume M., Mensink G.B.M. (2022). Overweight and obesity among adults in Germany—Results from GEDA 2019/2020-EHIS. J. Health Monit..

